# Outcome of elderly patients with acute promyelocytic leukemia: results of the German Acute Myeloid Leukemia Cooperative Group

**DOI:** 10.1007/s00277-012-1597-9

**Published:** 2012-10-23

**Authors:** Eva Lengfelder, Benjamin Hanfstein, Claudia Haferlach, Jan Braess, Utz Krug, Karsten Spiekermann, Torsten Haferlach, Karl-Anton Kreuzer, Hubert Serve, Heinz A. Horst, Susanne Schnittger, Carlo Aul, Beate Schultheis, Philipp Erben, Stephanie Schneider, Carsten Müller-Tidow, Bernhard Wörmann, Wolfgang E. Berdel, Cristina Sauerland, Achim Heinecke, Rüdiger Hehlmann, Wolf-Karsten Hofmann, Wolfgang Hiddemann, Thomas Büchner, for the German Acute Myeloid Leukemia Cooperative Group (AMLCG)

**Affiliations:** 1 Department of Hematology and Oncology, University Hospital Mannheim, Mannheim, Germany; 2 Munich Leukemia Laboratory (MLL), Munich, Germany; 3 Internal Medicine III, University Hospital Grosshadern, Ludwig-Maximilian-University Munich, Munich, Germany; 4 Department of Medicine A, Hematology–Oncology, University of Münster, Münster, Germany; 5 Department I of Internal Medicine, University of Cologne, Cologne, Germany; 6 Department of Hematology and Oncology, University of Frankfurt, Frankfurt, Germany; 7 Medical Department II, University of Kiel, Kiel, Germany; 8 St. Johannes Hospital, Duisburg, Germany; 9 Department of Hematology and Oncology, Marienhospital Herne, Ruhr University Bochum, Herne, Germany; 10 Department of Hematology and Oncology, Charité University Medicine Berlin, Campus Virchow-Klinikum, Berlin, Germany; 11 Institute of Biostatistics and Clinical Research, University of Münster, Münster, Germany; 12 III. Medizinische Klinik, Hämatologie und Onkologie, Universitätsmedizin Mannheim, Theodor-Kutzer-Ufer 1-3, 68167 Mannheim, Germany

**Keywords:** Acute promyelocytic leukemia, Elderly patients, Early death, Treatment

## Abstract

Despite improvement of prognosis, older age remains a negative prognostic factor in acute promyelocytic leukemia (APL). Reports on disease characteristics and outcome of older patients are conflicting. We therefore analyzed 91 newly diagnosed APL patients aged 60 years or older (30 % of 305 adults with APL) registered by the German AML Cooperative Group (AMLCG) since 1994; 68 patients (75 %) were treated in studies, 23 (25 %) were non-eligible, and 31 % had high-risk APL. Fifty-six patients received induction therapy with all-trans retinoic acid and TAD (6-thioguanine, cytarabine, daunorubicin), and consolidation and maintenance therapy. Treatment intensification with a second induction cycle (high dose cytarabine, mitoxantrone; HAM) was optional (*n* = 14). Twelve patients were randomized to another therapy not considered in this report. The early death rate was 48 % in non-eligible and 19 % in study patients. With the AMLCG regimen, 7-year overall, event-free and relapse-free survival (RFS) and cumulative incidence of relapse were 45 %, 40 %, 48 %, and 24 %, respectively. In patients treated with TAD–HAM induction, 7-year RFS was superior (83 %; *p* = 0.006) compared to TAD only, and no relapse was observed. In our registered elderly patients, we see a high rate of non-eligibility for treatment in studies and of high-risk APL. In patients who can undergo a curative approach, intensified chemotherapy is highly effective, but is restricted to a selection of patients. Therefore, new less toxic treatment approaches with broader applicability are needed. Elderly patients might be a particular target group for concepts with arsenic trioxide.

## Introduction

With current treatment strategies, 70 % to 80 % of patients with acute promyelocytic leukemia (APL) included in multicenter trials can be cured. Despite improvement of prognosis over the last two decades, older age remains a prominent negative prognostic factor [1].

Older APL patients seem to be as sensitive to specific APL therapy as younger individuals. It is generally assumed that in elderly APL patients a higher vulnerability to treatment-related toxicity causes an increased rate of treatment-related mortality. These observations led to recommendations to reduce the intensity of the chemotherapy combined with all-trans retinoic acid (ATRA) in older APL patients or to use less toxic strategies based on arsenic trioxide [1–4].

In larger studies, the incidence of APL patients aged over 60 and 70 years ranged from 9 % to 24 % and 3 % to 7 %, respectively, giving the impression that APL is relatively rare in older age [5–10]. Few publications have specifically reported the outcome in larger series of older patients. These studies included on average 15 % high-risk patients according to the Sanz Score suggesting that older APL patients present more frequently with low-risk features [2, 7, 11, 12].

A population-based study from the USA reported an early death (ED) rate of 24 % in APL patients aged 55 years or older. In the Swedish Adult Leukemia Registry, the ED rate of 105 unselected APL patients of all age groups was 29 %, and of patients over 60 years, it was 50 % [13, 14]. This is much higher than reported in treatment studies. It can therefore be assumed that a considerable proportion of elderly patients is not included in clinical trials.

We report here the characteristics and outcome of 91 APL patients over 60 years representing 30 % of all APL patients registered by the German AML Cooperative Group (AMLCG) from 1994 to 2011. A relatively high rate (31 %) was assigned to the high-risk group and 25 % were not eligible for inclusion in the studies. The treatment consisted of ATRA and chemotherapy. The design allowed individual intensification of the therapy.

## Design and methods

### Eligibility criteria and recruitment of patients

From December 1994 to November 2011, 305 adult patients with newly diagnosed APL have been registered by 60 centers participating in the studies of the AMLCG. From 1994 to November 2005, the patients were uniformly treated with the regimen of the German AMLCG [15]. Since December 2005, the patients were randomly assigned to the previous AMLCG protocol or to the strategy of the Spanish PETHEMA (German Clinical Trials Register, numbers DRKS00004313 and DRKS00004314). Eligibility criteria were newly diagnosed APL, cytogenetic or molecular confirmation of the diagnosis, age over 16 years without upper age limit, and written informed consent. Exclusion criteria were other antecedent or concomitant malignancy; cardiac failure NYHA III and IV; chronic renal failure with serum creatinine ≥2 mg/100 ml; severe liver disease; pneumonia with hypoxemia, sepsis, or other severe infection; uncontrolled life-threatening bleeding; and frailty. In accordance with the Declaration of Helsinki, the protocols were approved by the Research Ethics Board of each participating center. The confirmation of the diagnosis APL by morphological, cytogenetic, and/or molecular characterization of the leukemic cells in central laboratories was mandatory. All data of the patients were registered at the Institute of Biostatistics and Clinical Research, University of Münster, Germany.

**Fig. 1:**
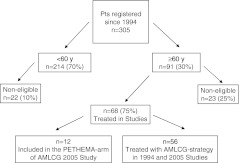
Flow chart separating according to age, eligibility, and treatment of the 305 APL patients registered by the German AMLCG. Ninety-one patients (30 %) were ≥60 years; of these, 68 (75 %) were treated in studies and 23 (25 %) were non-eligible

Ninety-one patients (30 %) (Fig. 1) were ≥60 years and 33 patients (11 %) ≥70 years. Of these, 68 patients (75 %) were eligible for inclusion in the study. The reasons for non-eligibility in the 23 remaining patients (25 %) were death before therapy (*n* = 5), reduced performance status/comorbidity according to the exclusion criteria (*n* = 11), and concomitant malignancy (*n* = 7). Fifty-six patients were treated with the AMLCG regimen, 47 patients in the first and nine patients in the second protocol. The 12 patients treated according to the PETHEMA strategy were not considered in this report to assure homogeneity of therapy.

### Design of the AMLCG regimen for elderly patients

Patients over 60 years received an age-adapted version of the AMLCG protocol with less intensive chemotherapy as the younger patients [15]. The induction therapy started with all-trans retinoic acid (ATRA) 45 mg/m^2^/day and simultaneous chemotherapy with TAD. The administration of the second induction cycle, HAM with high-dose cytarabine (ara-C), was at the discretion of the treating physician. Its application was mainly influenced by the assessment of the physician, whether the patient could tolerate the toxicity of two induction cycles. The start of HAM was scheduled at day 21 after the start of TAD and could be delayed in dependence on the individual tolerance of the first cycle. ATRA was continued until complete hematological remission (CR) or for a maximum of 90 days independent of the number of induction cycles. [TAD—6-thioguanine (6-TG) 100 mg/m^2^ orally every 12 h, days 3–9, ara-C 100 mg/m^2^ by continuous intravenous (i.v.) infusion, days 1–2, thereafter 100 mg/m^2^ as i.v. infusion every 12 h, days 3–8, daunorubicin (DNR) 60 mg/m^2^ i.v. infusion, days 3–5; HAM—ara-C 1 g/m^2^ (instead of 3 g/m^2^ as in younger patients) i.v. infusion every 12 h, days 1–3, mitoxantrone 10 mg/m^2^ i.v. infusion, days 3–5].

Supportive treatment for coagulopathy, oral antimicrobial prophylaxis as well as diagnosis and management of the APL differentiation syndrome were performed according to international standards [15].

Consolidation therapy with one cycle TAD started 2 to 4 weeks after CR followed by 3-year monthly maintenance therapy consisting of ara-C 200 mg/m^2^/day s.c. over 5 days combined with either DNR 45 mg/m^2^, days 3 and 4 (course 1), or 6-TG 200 mg/m^2^/day over 5 days (course 2 and 4), or cyclophosphamide 1 g/m^2^ i.v., day 3 (course 3). The sequence was restarted with DNR (course 5). After a cumulative DNR dose of 540 mg/m^2^, DNR was replaced by 6-TG. Dose adaptations were performed as described elsewhere [15].

Bone marrow aspiration for morphological evaluation and molecular monitoring by RT–PCR was mandatory after induction and consolidation, and was recommended every 3 months during maintenance therapy.

### Definitions and endpoints

CR was defined according to the National Cancer Institute criteria [16] and molecular remission as negative nested RT–PCR for PML–RARA (sensitivity 10^−4^). Molecular relapse had to be confirmed by nested RT–PCR in two marrow samples taken 2 to 4 weeks apart. In eligible patients, ED was defined as death between start of therapy and achievement of CR, and in non-eligible patients as death within 30 days after diagnosis [13]. Overall survival (OS) was calculated from the start of induction therapy until death for eligible patients, and from diagnosis until death in non-eligible patients, event-free survival (EFS) from the start of therapy until non-achievement of remission or relapse or death, and relapse-free survival (RFS) from the date of CR until molecular or hematological relapse or death in remission, whichever occurred first. The cumulative incidence of relapse (CIR) was calculated from the date of CR until molecular or hematological relapse taking into account death in CR as competing risk.

### Statistical analysis

Comparison of the CR rates and categorical variables was evaluated by Fisher’s exact test. Continuous variables were compared using the Mann–Whitney *U* test. All *p* values reported are two-sided. Distributions of time-to-event variables were estimated by the Kaplan–Meier method [17]. Comparisons were based on the log-rank test. Cumulative incidences were calculated according to Cooley et al. [18]. Differences in the rates of CIR were calculated with the Gray test [19]. Analysis of the reported data was in November 2011. All calculations were done using SAS software (version 9.2) licensed to the University of Münster, Germany.

## Results

### Patient characteristics

The initial characteristics of all 91 registered patients were separated according to non-eligibility (*n* = 23), treatment within the study (*n* = 68), and treatment with the AMLCG regimen (*n* = 56) (Table 1). In all cases, APL was genetically confirmed. The rate of high-risk patients according to the Sanz Score was 31 %. Sixty percent of patients had the BCR3 transcript (short variant), and 50 % had additional cytogenetic abnormalities in combination with the translocation t(15;17). Besides a higher rate of non-eligible females (*p* = 0.002), there was no statistical difference of the initial patient characteristics between the four groups.

**Table 1:** Baseline patients’ characteristics

	All registered patients			Non-eligible patients			In-study patients			*p* value^a^	Treatment with AMLCG regimen			*p* value^b^
No. of patients	91			23			68				56			
Characteristics	Median (range)	No.	(%)	Median (range)	No.	(%)	Median (range)	No.	(%)		Median (range)	No.	(%)	
Age (years)	67 (60–87)			67 (60–83)			67 (60–87)			0.5	67 (60–83)			0.4
60–69		58	(64)		13	(57)		45	(66)	0.5		38	(68)	0.4
70 and older		33	(36)		10	(43)		23	(34)			18	(32)	
Gender														
Male		42	(46)		4	(17)		38	(56)	0.002		33	(59)	0.001
Female		49	(54)		19	(83)		30	(44)			23	(41)	
WBC, ×10^9^/L	2.2 (0.4–103)			2.2 (0.6–103)			2.4 (0.4–100)			0.9	2.9 (0.4–71.8)			0.9
Less than 5		49	(58)		10	(59)		39	(57)	0.6		31	(55)	0.7
5–10		10	(12)		3	(18)		7	(10)			7	(13)	
More than 10		26	(30)		4	(23)		22	(32)			18	(32)	
Platelets, ×10^9^/L	26 (3–192)			21 (2–192)			27 (3–177)			0.7	26 (3–177)			0.7
40 or more		28	(33)		5	(29)		23	(34)	0.8		17	(30)	1.0
Less than 40		56	(77)		12	(71)		44	(66)			39	(70)	
Hemoglobin, g/L	9.4 (4.3–15.3)			9.2 (4.3–11.2)			9.4 (5.3–15.3)			0.5	9.4 (5.3–15.3)			0.5
10 or less		31	(39)		6	(40)		25	(38)	1.0		21	(38)	1.0
More than 10		49	(61)		9	(60)		40	(62)			34	(62)	
Morphology														
Hypergranular		60	(66)		17	(74)		43	(63)	0.4		34	(61)	0.3
Microgranular (M3v)		31	(34)		6	(26)		25	(37)			22	(39)	
Cytogenetics														
t(15;17)		40	(50)		9	(50)		31	(50)	1.0		28	(52)	1.0
t(15;17) and others^c^		40	(50)		9	(50)		31	(50)			26	(48)	
PML/RARA isoform														
BCR1/BCR2		33	(40)		7	(37)		26	(41)	0.8		20	(38)	1.0
BCR3		49	(60)		12	(63)		37	(59)			32	(62)	
Risk group (Sanz Risk Score)														
Low		23	(27)		4	(25)		19	(28)	0.4		15	(27)	0.5
Intermediate		36	(42)		9	(56)		27	(40)			23	(41)	
High		25	(31)		3	(19)		22	(32)			18	(32)	

^a^ *p* compares non-eligible and all in-study patients

^b^ *p* compares non-eligible patients and patients treated with the AMLCG-regimen

^c^t(15;17) and other chromosomal aberrations

In the 56 patients treated with the AMLCG regimen, ATRA was started simultaneously with TAD in 30 patients and before TAD in 26 patients (median 6 days, range 1 to 12). Fourteen patients (25 %) received the second induction cycle (HAM). In five of these patients, the CR criteria were reached before the start of HAM. The median time between start of TAD until HAM was 35 days (range 22 to 55). Retrospectively analyzed, the relevant characteristics of patients treated with TAD–HAM showed no significant difference to patients alive after TAD and treated with TAD induction only. Notably, there was no statistical difference of the duration of ATRA therapy in both groups (*p* = 0.4), of the percentage of patients below and above 70 years (*p* = 0.6), and of patients with high versus low/intermediate risk according to the Sanz Score (*p* = 0.5) (Table 2).

**Table 2:** Characteristics of patients with TAD–HAM vs. TAD induction therapy

Induction cycles	TAD–HAM	TAD only	*p* value
Patients alive after TAD induction course	*n* = 14	*n* = 32	
Median age (range)	66 (61–70)	66 (60–80)	0.6
Gender, ratio (male/female)	2.5	1.1	0.3
WBC count median (range) ×10^9^/L	1.4 (0.4–26.1)	2.4 (0.4–71.8)	0.6
Initial WBC count less than 10 × 10^9^/L: *n*, (%)	12 (86)	23 (72)	0.5
Initial WBC count 10 × 10^9^/L or more: *n*, (%)	2 (14)	9 (28)	
Low risk^a^: *n*, (%)	8 (57 %)	7 (22 %)	0.07
Intermediate risk^a^: *n*, (%)	4 (29 %)	16 (50 %)	
High risk^a^: *n*, (%)	2 (14 %)	9 (28 %)	
Fever/infection WHO grade 3 or more during course 1: *n*, (%)	5 (36)	15 (47)	0.5
APL differentiation syndrome (course 1): *n*, (%)	4 (29)	9 (28)	1.0
Days of ATRA; median (range)	51 (22–90)	38 (20–89)	0.4
Patients younger than 70 years (*n* = 34)	11 (32 %)	23 (68 %)	0.6
Patients 70 years or older (*n* = 12)	3 (25 %)	9 (75 %)	
RFS at 7 years	83 [60;100]	35 [16;54]	0.01
CIR at 7 years	0	34 [20;56]	0.03

*WBC count* white blood cell count, *ATRA* all-trans retinoic acid, *RFS* relapse-free survival, *CIR* cumulative incidence of relapse

^a^According to Sanz Risk Score

### Response to induction therapy

The results of induction therapy of all patients and separated according to initial WBC count and age below and above 70 years are shown in Table 3. Hematological CR was achieved in 82 % of patients. The median time to CR was 37 days (range 22 to 55) in patients with TAD only, and 70 days (24 to 89) in patients treated with TAD–HAM. No resistance against therapy was observed. ED occurred in 18 % of patients between days 2 and 19 after start of induction therapy. No patient died from ED after TAD–HAM. The ED rate was higher in the high WBC group (*p* = 0.009) and in patients over 70 years (*p* = 0.06). Causes of ED were bleeding, multiorgan failure, and sepsis (Table 4). Three patients died in CR on days  33, 50, and 73, respectively, after start of induction therapy from cardiac failure (*n* = 1) or for unknown reasons (*n* = 2). Molecular remission was documented in 22 of 33 (67 %) patients after induction.

**Table 3:** Treatment results

	All patients	WBC less than 10 × 10^9^/L	WBC 10 × 10^9^/L or more	*p* value	Age 60–69 years	Age 70 years or older	*p* value
							
	*n* = 56	*n* = 38	*n* = 18		*n* = 38	*n* = 18	
							
	*n* (%)	*n* (%)	*n* (%)		*n* (%)	*n* (%)	
CR (%)	46 (82)	35 (92)	11 (61)		34 (89)	12 (67)	
Early death (%)	10 (18)	3 (8)	7 (39)	0.009	4 (11)	6 (33)	0.06

OS at 7 years (%)	45 [30;61]	55 [37;73]	26 [5;47]	0.002	54 [37;72]	25 [1;75]	0.048
EFS at 7 years (%)	40 [26;54]	53 [35;70]	15 [0;33]	0.0001	46 [49;63]	28 [4;52]	0.1
RFS at 7 years (%)	48 [32;65]	57 [38;76]	24 [0;52]	0.008	51 [32;70]	42 [9;75]	0.8
CIR at 7 years (%)	24 [14;41]	13 [05;32]	58 [34;98]	0.004	26 [14;48]	19 [5;65]	0.7

*WBC* white blood cell count, *CR* complete hematological remission, *OS* overall survival, *EFS* event-free survival, *RFS* relapse-free survival, *CIR* cumulative incidence of relapse

**Table 4:** Time and cause of death

Age at diagnosis (years); gender	Bone marrow morphology	WBC count at diagnosis (×10^9^/L)	Time of death (days counted from the first day of induction therapy)	Cause of death
Early death *n* = 10				
71; f	M 3 v	46.0	2	Multiorgan failure
78; f	M 3 v	23.0	3	Multiorgan failure
66; m	M 3 v	26.9	5	Bleeding (cerebral)
75; f	M 3	15.6	7	Bleeding (pulmonal)
83; m	M 3 v	15.4	9	Multiorgan failure
83; m	M 3	1.0	12	Infection/sepsis
67; m	M 3 v	42.1	15	Infection/sepsis
67; m	M 3 v	19.0	15	Bleeding (pulmonal)
82; f	M 3	12.0	17	Infection/sepsis
68; m	M 3	59.0	19	Multiorgan failure
Death in CR after induction or consolidation *n* = 5				
80; m	M 3 v	1.2	33	Cardiac failure
68; f	M 3 v	7.8	50	Not reported, in CR of APL
65; f	M 3 v	45	73	Multiorgan failure
64; m	M 3	0.4	74	Sepsis
61;f	M 3	9.4	77	Sepsis
Death in CR during follow-up *n* = 6				
65; f	M 3	22.0	237	Sepsis in cytopenia after maintenance
75; m	M 3 v	0.9	888	Not reported, in CR of APL
62; m	M3	0.6	1637	After MDS and allo. transpl., in CR of APL
75; f	M 3	0.5	1896	Not reported, in CR of APL
75; m	M 3	2.5	2263	Poor performance status, in CR of APL
69; m	M 3 v	19.3	3359	Colon carcinoma, in CR of APL
Death after relapse *n* = 8				
75; f	M 3	1.7	351	First relapse
60; f	M 3	1.2	788	Third relapse
66; f	M3 v	71.8	895	Second relapse
64; m	M 3 v	41.0	1037	Second relapse
65; f	M 3 v	7.1	1418	Second relapse
61; m	M 3	30.8	1638	First relapse
71; f	M 3	25.1	1821	Second relapse
70; m	M 3	0.5	2140	Second relapse and GIST

*WBC count* white blood cell count, *CR* complete remission, *MDS* myelodysplastic syndrome, *allo*. *transpl*. allogeneic transplantation, *GIST* gastrointestinal stromal tumor

The APL differentiation syndrome was diagnosed in 25 % of patients between days 2 and 29 after the start of induction therapy. Other adverse effects WHO grade ≥3 were fever or infection in 43 % of patients (45 % in patients treated with TAD only and 36 % in patients with TAD/HAM); cardiac failure was observed in 16 %, diarrhea in 13 %, hepatotoxicity in 11 %, and hemorrhage in 7 % of patients.

### Post-remission outcome

Consolidation therapy with TAD was administered in 36 of the 43 (84 %) living patients and omitted in seven cases (16 %) due to severe side effects during induction. Two patients died after consolidation from sepsis in neutropenia on days 74 and 77, respectively, after start of induction therapy (Table 4). After consolidation therapy, 31 of 33 (94 %) tested patients were in molecular remission. Maintenance therapy was started in 71 % of the living patients (29 of 41), corresponding to 57 % of the TAD–HAM group and to 65 % of the TAD-only group. It was prematurely terminated between course 1 and 8 in eight cases (20 %) resulting in completion of maintenance therapy in 50 % of the TAD–HAM patients and of 44 % of the TAD-only group.

**Fig. 2:**
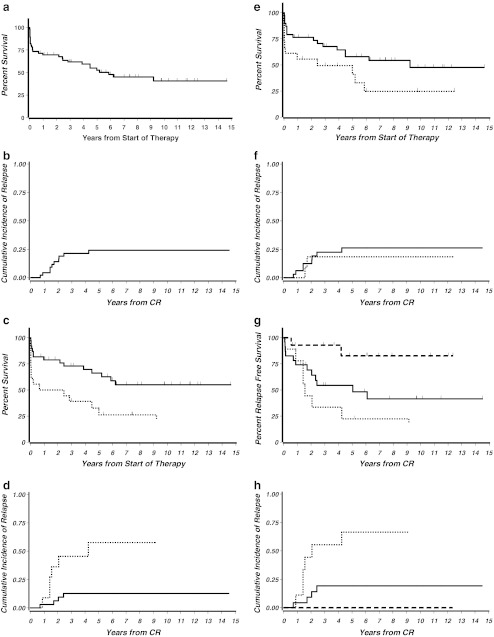
**a** OS of all patients treated with the AMLCG regimen (*n* = 56). Median observation time was 7.4 years. The OS was 45 % at 7 years. **b** CIR of all patients treated with the AMLCG regimen who entered remission (*n* = 46). CIR of these patients was 24 % at 7 years. **c** OS of the 56 patients treated with the AMLCG regimen separated according to WBC counts less than and ≥10 × 10^9^/L. OS was significantly superior in patients with WBC counts <10 × 10^9^/L (*p* = 0.002). *Bold line*: OS of patients with initial WBC counts <10 × 10^9^/L (*n* = 38) was 55 % at 7 years; *dotted line*: OS of patients with WBC counts ≥10 × 10^9^/L (*n* = 18) was 26 % at 7 years. **d** CIR of the 46 patients who entered remission after treatment with the AMLCG regimen separated according to WBC counts less than and ≥10 × 10^9^/L. CIR was significantly superior in patients with WBC counts <10 × 10^9^/L (*p* = 0.004). *Bold line*: CIR of patients with initial WBC counts <10 × 10^9^/L (*n* = 35) was 13 % at 7 years; *dotted line*: CIR of patients with WBC counts ≥10 × 10^9^/L (*n* = 11) was 58 % at 7 years. **e** OS of the 56 patients treated with the AMLCG regimen separated according to age 60 to 69 and ≥70 years. OS was significantly shorter in advanced age (*p* = 0.048). *Bold line*: OS of patients 60 to 69 years of age (*n* = 38) was 54 % at 7 years; *dotted line*: OS of patients >70 years (*n* = 18) was 25 % at 7 years. **f** CIR of the 46 patients who entered remission after treatment with the AMLCG regimen separated according to age 60 to 69 and ≥70 years. There was no significant difference of CIR in both groups (*p* = 0.7). *Bold line*: CIR of patients 60 to 69 years of age (*n* = 34) was 26 % at 7 years; *dotted line*: CIR of patients ≥70 years (*n* = 12) was 19 % at 7 years. **g** RFS of patients treated with TAD–HAM or with only one induction course, TAD. In the group with TAD alone, the patients were additionally separated according to initial WBC counts less than and ≥10 × 10^9^/L. The outcome was significantly improved after TAD–HAM (*p* = 0.006) as compared to TAD induction alone. *Bold line*: RFS of patients with initial WBC counts <10 × 10^9^/L (*n* = 23) and treatment with only one induction cycle (TAD) was 41 % at 7 years; *dotted line*: RFS of patients with WBC counts ≥10 × 10^9^/L (*n* = 9) and treatment with only one induction cycle was 22 % at 7 years; *broken line*: RFS of the patients treated with TAD–HAM induction (*n* = 14) was 83 % at 7 years. **h** CIR of patients treated with the AMLCG regimen separated according to double induction therapy with TAD–HAM and with only one induction course, TAD. In the group with TAD alone, the patients were additionally separated according to initial WBC counts less than and ≥10 × 10^9^/L. There was a significantly lower CIR after TAD–HAM (*p* = 0.002) as compared to TAD induction alone. *Bold line*: CIR of patients with initial WBC counts <10 × 10^9^/L (*n* = 23) and treatment with only one induction cycle (TAD) was 19 % at 7 years; *dotted line*: CIR of patients with WBC counts ≥10 × 10^9^/L (*n* = 9) and treatment with only one induction cycle was 67 % at 7 years; *broken line*: CIR of the patients treated with TAD–HAM induction (*n* = 14) was 0 % at 7 years

The median follow-up of the 56 patients treated with the AMLCG regimen was 7.4 years (1 day to 14.6 years). The 7-year OS was 45 %, EFS 40 %, RFS 48 %, and CIR 24 % (Table 3, Fig. 2a, b). Ten relapses occurred. All were hematological relapses of the bone marrow, in two cases preceded by molecular relapse. No extramedullary relapse was observed. Of the 29 total deaths, 10 were due to ED, eight due to first or later relapse, and 11 patients died in CR. Approximately 50 % of these deaths in CR were probably therapy-related after induction, consolidation, and during maintenance therapy, and 50 % died for other reasons during long-term follow-up (Table 4). Secondary malignancies were observed in six patients (11 %) (MDS/secondary AML, *n* = 3; non-Hodgkin lymphoma, *n* = 1; GIST, *n* = 1; colon carcinoma, *n* = 1).

Patients with initial WBC counts ≥10 × 10^9^/L had a significantly inferior outcome concerning OS (*p* = 0.002), EFS (*p* = 0.0001), RFS (*p* = 0.008), and CIR (*p* = 0.004). There was no significant difference between RFS and CIR of intermediate and low-risk patients according to the Sanz Score, respectively [11]. Advanced age ≥70 years was associated with a shorter OS as compared to age <70 years (*p* = 0.048). However, when CR was reached, RFS (*p* = 0.8) and CIR (p = 0.7) did not differ significantly (Table 3, Fig. 2c–f).

Patients who achieved CR after TAD–HAM had a superior RFS of 83 % (*p* = 0.006) and a better CIR (*p* = 0.002), as no relapse was observed in the TAD–HAM group. For comparison, in the patients treated with TAD induction only, the RFS was 35 % and the CIR 34 %. Separated according to initial WBC counts less than or ≥10 × 10^9^/L, the rates of RFS and CIR after TAD induction only were 41 % and 19 %, and 22 % and 67 %, respectively (Fig. 2g, h).

### Outcome of the non-eligible patients

**Fig. 3:**
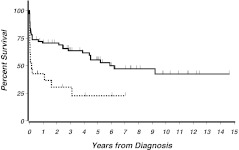
OS of study patients compared to non-eligible patients. *Bold line*: OS of all patients treated in studies (*n* = 68); *dotted line*: OS of the non-eligible patients (*n* = 23) (*p* = 0.002). The non-eligible patients who survived longer than 2 years had received standard-like therapy

The 7-year OS of all 91 registered patients was 41 % [53;29]. Figure 3 separately shows the OS of eligible (*n* = 68) and non-eligible (*n* = 23) patients. In the non-eligible group, 48 % (*n* = 11) of patients died from ED after a median time of 9 days (range 2–22) as compared to 19 % ED (*n* = 13) in the 68 study patients (AMLCG or PETHEMA regimens). Antecedent malignancy (*n* = 3) and severe pneumonia requiring mechanical ventilation (*n* = 1) were reasons for exclusion of the four non-eligible patients, who survived longer than 2 years (Fig. 3). These patients received ATRA and anthracycline-based therapy outside of the study.

## Discussion

The data of the present study show that APL is curable in a considerable proportion of older patients. The present study differs from other reports. We found a considerable rate of older patients (25 %) not eligible for treatment with standard therapy leading to a positive selection of study patients and a high rate of patients with high-risk profile (31 %). Less intensive chemotherapy during induction was associated with an increased relapse rate and reduced RFS.

Concerning the high rate of non-eligibility of 25 %, it may be assumed that the true exclusion rate is even higher because the registration of non-eligible patients was not performed with the same stringency at all centers [20]. Remarkably, besides an unexplained higher rate of exclusions in females than in men, there was no significant difference in the initial patients’ characteristics between the eligible and non-eligible patients. In particular, there was no difference in the distribution of risk groups (Table 1). These data suggest that the non-eligibility of older patients is probably more determined by multimorbidity and performance status than by APL-specific factors. The median survival of the non-eligible patients was only 2 months, and longer survival was restricted to patients who unexpectedly improved and were later able to receive standard-like therapy (Fig. 3).

Forty-eight percent of the non-eligible and 19 % of the 68 patients included in the studies died within 22 days after diagnosis or after start of therapy. This corresponds to an ED rate of 26 % of all 91 registered patients. The results of the 56 patients, treated with the AMLCG regimen, show an increased rate of ED in patients with high WBC counts (*p* = 0.009) and with advanced age (Table 3). For comparison, in our 142 APL patients younger than 60 years and treated with the AMLCG-type chemotherapy, the ED rate was only 8 % [15]. Causes of ED differed with age. Besides fatal hemorrhage in 30 %, multiorgan failure and sepsis caused 70 % of the ED in the elderly patients, which is possibly a consequence of the reduced tolerance of the chemotherapy. In our younger patients, bleeding was the cause of death in 64 % of ED and sepsis in only 9 % [15]. These data show that the high ED rate is a great and unresolved problem with high impact on the outcome of older patients, as also reported by others [13, 14, 20–22].

The 7-year OS of patients treated with the AMLCG regimen was 45 % and of all registered patients 41 %, compared to more than 80 % in younger APL patients [3, 9, 15, 23]. The percentage of 30 % registered elderly patients and their outcome are comparable to recently published data of a population-based study from the USA. This analysis reported the registration of 32 % APL patients older than 60 years among 1,397 APL cases and a 5-year survival rate below 40 % in cases diagnosed between the years 2000 and 2008 [24].

The OS of our study patients was significantly shorter in patients with high WBC counts and advanced age over 70 years (Fig. 2a, c, e). It was much better, however, compared to older patients with other AML subtypes, who reached cure in about 15 % [25]. Twenty-four percent of the patients, who had achieved remission, died in CR. In approximately 50 % of these patients, the chemotherapy-related toxicity had an influence on the fatal outcome (Table 4). Twenty percent of patients died in first or later relapse. These data demonstrate that three main reasons had an impact on the fatal outcome: the high ED rate, the chemotherapy-related toxic effects followed by death in CR, and the high relapse rate. Similar observations were made by others [2, 3, 7, 20, 21]. But due to differences of the risk profile of the included patients and of the treatment regimens, a direct comparison with other studies is not possible.

The results further show that our older patients who received intensified chemotherapy with HAM benefited from this approach, reflecting the results of younger patients (Fig. 2g, h) [15, 26]. An inferior outcome after only one induction course (TAD) was seen in low-risk/intermediate and high-risk patients. These patients received a relatively low cumulative daunorubicin dose of 360 mg/m^2^ during induction and consolidation therapy and no high dose ara-C and no mitoxantrone. The beneficial prognostic influence of higher doses of chemotherapy combined with ATRA is also supported by other reports. The Spanish PETHEMA reported an excellent outcome in older APL patients with ATRA and a relatively high cumulative dose of anthracyclines (68 to 100 mg/m^2^ idarubicin and 50 mg/m^2^ mitoxantrone) [12, 27]. However, in a historical comparison of the Italian studies, the reduction of poly-chemotherapy cycles had beneficial effects on survival due to reduction of toxicity [2, 22]. In younger Italian low-risk/intermediate risk patients treated with the PETHEMA-type concept, the expanded application of ATRA during anthracycline-based consolidation therapy improved the outcome despite omission of the non-anthracycline part of chemotherapy [23]. In elderly APL patients, it would be of particular interest in how far the amount of chemotherapy could be reduced when prolonged ATRA was administered. Currently, no general recommendation can be given which dose or modification of chemotherapy and ATRA is optimal.

Our observations suggest that a considerable proportion of older APL patients with poor prognosis are excluded from treatment in studies and indicate that the results of clinical trials do not reflect the true outcome of the entire older APL population. In elderly patients who can undergo a curative approach, intensified chemotherapy is highly effective, but remains restricted to a selection of patients. Therefore, new less toxic but efficient treatment approaches with broader applicability are needed in elderly APL. As suggested by recent reports, elderly patients may represent a particular target group to replace chemotherapy by arsenic trioxide, which should be addressed in future trials [28–31]. Furthermore, molecular markers might contribute to better adapted treatment in genetically determined APL risk groups also in the elderly patients [32].

## Appendix

The following institutions and clinicians participated in the study: University Hospital, Aachen (T. Ittel); Municipal Hospital Augsburg (C. Schmid); Municipal Hospital, Bad Saarow (H. Fuß); Municipal Hospital Neukölln, Berlin (A. Mayr, A. Grüneisen); University Hospitals Charité, Berlin (R. Arnold, B. Dörken, G. Maschmeyer, A. Trittin); University Hospital Benjamin Franklin, Berlin (M. Notter, E. Thiel); University Hospital Robert Rössle, Berlin (W.D. Ludwig, D. Schöndube); Municipal Hospital, Bielefeld (A. J. Weh, A. Zumsprekel); Knappschaft Hospital, Bochum (C. Teschendorf, M. Stechstor); Knappschaft Hospital, Bottrop (G. Trenn); Diakonissen Hospital, Bremen (G. Kessler, Th. Wolff); Municipal Hospital St. Jürgenstraße, Bremen (B. Hertenstein, H. Thomssen, A. Peyn); Municipal Medical Center, Braunschweig (B. Wörmann); St. Johannes Hospital, Dortmund (H. Pielken, H. Hindahl); University Hospital, Düsseldorf (A. Heyll); Municipal Hospital, Düren (M. Flaßhove, J. Karow); St. Johannes Hospital, Duisburg (C. Aul, C. Giagounidis); St. Antonius Hospital, Eschweiler (R. Fuchs, F. Schlegel); Tumorklinik, Essen (S. Seeber); Evangelian Hospital, Krankenhaus Essen-Werden, (Ch. Tirier); St. Joseph-Hospital, Gelsenkirchen (G. Meckenstock, G. Giagounidis); University Hospital, Göttingen (D. Haase, F. Griesinger); Municipal Hospital, Gütersloh (C. Gropp, R. Depenbusch); Municipal Hospital, Hagen (H. Eimermacher, W. Lindemann); Municipal Hospital Martha-Maria, Halle (W. Schütte, U. Haak); General Hospital Altona, Hamburg (D. Braumann); Evangelian Hospital, Hamm (L. Balleisen); Municipal Hospital Siloah, Hannover (H. Kirchner); District Hospital, Herford (J.G. Lange, U. Schmitz-Hübner); Marien Hospital, Herne (B. Schultheis); Municipal Hospital, Idar-Oberstein (A. Fauser); University Hospital, Jena (H.-J. Fricke); Municipal Hospital, Kassel (M. Wolf, B. Ritter); Westpfalz Hospital, Kaiserslautern (H. Link); University Hospital II, Kiel (M. Kneba, H.A. Horst); University Hospital, Köln (M. Hallek, P. Staib, K.A. Kreuzer); Municipal Hospital, Köln (A. Dormann); Municipal Hospital, Krefeld (Th. Frieling, M. Planker); Hospital Lippe-Lemgo, Lemgo (F. Hartmann, H. Middeke); Dreifaltigkeit-Hospital, Lippstadt ( K.A. Jost); Municipal Hospital, Leverkusen (N. Niederle); University Hospital, Lübeck (Th. Wagner); Municipal Hospital South-Lübeck, Lübeck (S. Fetscher, J. Schmielau); Municipal Hospital, Ludwigshafen (M. Uppenkamp, M. Hoffmann); University Hospital, Mannheim (R. Hehlmann, E. Lengfelder, W.-K. Hofmann); St. Walburga Hospital, Meschede (M. Schwonzen); Maria-Hilf-Hospital, Mönchengladbach (D. Graeven, D. Kohl); Municipal Hospital Harlaching, München (M. Hentrich, X. Schiel); University Hospital Innenstadt, München (B. Emmerich, R. Dengler, B. Schlag); University Hospital Großhadern, München (W. Hiddemann, K. Spiekermann, J. Braess); Municipal Hospital Neuperlach, München (K. Nibler, D. Fleckenstein); Hospital Barmherzige Brüder, Regensburg (M. Schenk); University Hospital, Münster (T. Büchner, W. E. Berdel, U. Krug, C. Müller-Tidow); Municipal Hospital, Osnabrück (J. Hartlapp, T. Hegge, R. Peceny); Paracelsus Hospital, Osnabrück (O. Koch, G. Innig); Municipal Medical Center, Passau (Th. Südhoff, Th. Wagner); University Hospital, Regensburg (A. Reichle, R. Andreesen); Municipal Hospital, Siegen (W. Gassmann, T. Gaska); Ammerland-Klinik, Westerstede (D. Kohl); Dr. Horst Schmitt Hospital, Wiesbaden (N. Frickhofen, H-G. Fuhr); Heinrich Braun Hospital, Zwickau (U. Kreibich, G. Schott, S. Sommer, W. Zschille).
